# Development of a defined medium for the heterotrophic cultivation of *Metallosphaera sedula*

**DOI:** 10.1007/s00792-024-01348-0

**Published:** 2024-07-26

**Authors:** Viktor Laurin Sedlmayr, Maximilian Luger, Ernst Pittenauer, Martina Marchetti-Deschmann, Laura Kronlachner, Andreas Limbeck, Philipp Raunjak, Julian Quehenberger, Oliver Spadiut

**Affiliations:** 1 TU Wien, Institute of Chemical, Environmental and Bioscience Engineering, 1060 Vienna, Austria; 2 TU Wien, Institute of Chemical Technologies and Analytics, 1060 Vienna, Austria

**Keywords:** *Metallosphaera sedula*, Defined cultivation medium, Casamino Acids, Amino acids, Media development, Tetraether lipids

## Abstract

The heterotrophic cultivation of extremophilic archaea still heavily relies on complex media. However, complex media are associated with unknown composition, high batch-to-batch variability, potential inhibiting and interfering components, as well as regulatory challenges, hampering advancements of extremophilic archaea in genetic engineering and bioprocessing. For *Metallosphaera sedula*, a widely studied organism for biomining and bioremediation and a potential production host for archaeal ether lipids, efforts to find defined cultivation conditions have still been unsuccessful. This study describes the development of a novel chemically defined growth medium for *M. sedula*. Initial experiments with commonly used complex casein-derived media sources deciphered Casamino Acids as the most suitable foundation for further development. The imitation of the amino acid composition of Casamino Acids in basal Brock medium delivered the first chemically defined medium. We could further simplify the medium to 5 amino acids based on the respective specific substrate uptake rates. This first defined cultivation medium for *M. sedula* allows advanced genetic engineering and more controlled bioprocess development approaches for this highly interesting archaeon.

**Supplementary Information:**

The online version contains supplementary material available at 10.1007/s00792-024-01348-0.

## Introduction

As genetic systems and cultivation techniques have improved over the past years, extremophilic archaea are becoming more relevant in various fields of industrial biotechnology (Crosby et al. 2019; Rastädter et al. 2023). Extremophilic archaea hold immense promise as production hosts for lipids, stable enzymes and carotenoids (Chambers and Patrick 2015; Rastädter et al. 2020). The pH, temperature and salt conditions required for growth reduce the sterility requirements, and their persistence in extreme environments allows for applications in bioremediation and biomining (Quehenberger et al. 2017; Straub et al. 2018; Pfeifer et al. 2021). Underlining their growing industrial importance, several defined cultivation media have been developed to cultivate various thermophilic archaea, including species of the order *Sulfolobales* (Park and Lee 2000; Postec et al. 2005; Quehenberger et al. 2019).

Defined cultivation media have a precisely specified composition of all components, using pure chemicals in distinct proportions (Zhang and Greasham 1999). In contrast, complex media commonly contain hydrolysates or extracts of microbial biomass or by-products of the livestock- and crop-industry (Van Der Valk et al. 2010). Their composition is generally unknown and batch-dependent, and the presence of growth-inhibiting and interfering substances can occur (Park and Lee 2000). Therefore, defined media are required and wanted: in genetic engineering, defined media are necessary to control growth conditions precisely and facilitate mutant selection, as complex media may incorporate contaminants that interfere (McCarthy et al. 2018). Also, defined media are preferred in biomanufacturing to ensure batch-to-batch consistency and simplify regulatory documentation (Matthews et al. 2018). Here, the Quality-by-design guidelines are essential, as defined media allow consistent product quality and can improve understanding of the process during the development of a fermentation process (Quehenberger et al. 2017). Still, complex carbon and nitrogen sources, like N-Z-Amine, Tryptone, and Casein Hydrolysate, often in combination with sugars, are widely used to cultivate *Sulfolobales* based on the medium developed by Brock et al. (1972). This can be attributed to limited cell densities on defined media since cells must synthesize all cellular components from a few simple substrates.

*Metallosphaera sedula*, a member of *Sulfolobales*, is a facultative autotroph thermophile that thrives in low pH environments (Huber et al. 1989). In 2007, it was the host organism for the groundbreaking discovery of the 3-Hydroxypropionate/4-Hydroxybutyrate (3HP/4HB) cycle, a CO_2_ fixation pathway found exclusively in *Thermoproteota* (Berg et al. 2007, 2010). Populating metal-rich sulfuric fields, it has served as a model organism to study genes involved in metal oxidation and metal resistance of extremophilic prokaryotes (Auernik et al. 2008; Maezato et al. 2012; McCarthy et al. 2014). *M. sedula* can obtain energy from a variety of electron donors, like inorganic sulfur compounds, ferrous iron, uranium ores, and molecular hydrogen (Auernik and Kelly 2010a; Maezato et al. 2012; McCarthy et al. 2014; Wheaton et al. 2016; Milojevic et al. 2019). The ability of chemolithoautotrophic growth, where valuable metals, like Cu or Fe, can be extracted and solubilized from low-grade ores, has attracted significant interest in the field of biomining (Maezato et al. 2012; Abashina and Vainshtein 2023). Additionally, resistance against toxic substances and metal-oxidizing properties make *M. sedula* a very promising candidate in decontamination processes (Kavamura and Esposito 2010; Işıldar et al. 2019).

Besides its chemolithoautotrophic metabolism, *M. sedula* can be cultivated heterotrophically (Huber et al. 1989). This versatility makes *M. sedula* available for genetic engineering, as genetic transformation and subsequent selection are commonly performed on solid plates using organic C-sources. In the case of *Sulfolobales*, the majority of genetic systems use auxotrophies, most commonly uracil auxotrophies, for mutant selection (Lewis et al. 2021). *M. sedula* can grow on complex protein-based media sources (Beef Extract, Casamino Acids (CasA), Peptone, Tryptone, and Yeast Extract) but is not able to metabolize sugars (Huber et al. 1989). Additionally, an incomplete tricarboxylic acid and glyoxylate cycle pose a metabolic burden upon *M. sedula* during heterotrophic cultivations (Estelmann et al. 2011; Hawkins et al. 2014). Over the past years, several efforts have been made in defined media development, including formulations containing all 20 proteinogenic amino acids (AAs) and various combinations of AAs; however, all examined compositions did not allow biomass growth (Huber et al. 1989; McCarthy et al. 2018). This situation currently complicates handling with genetically engineered *M. sedula* strains. McCarthy et al. described the generation of the uracil auxotroph strain PBL4001, where successful selection could only be achieved after cumbersome serial passaging and depletion of uracil traces contained in the Tryptone-based cultivation medium.

*M. sedula* can be envisioned as an exciting production host for archaeal ether lipids, which are currently under investigation for utilization as lipid carriers (Santhosh and Genova 2023). Monolayer-spanning tetraether lipids have recently been associated with the formation of a hexagonal H_II_ phase in mRNA lipid nanoparticle delivery (Sedlmayr et al. 2024). So far, no detailed analysis of the lipid composition of *M. sedula* can be found in the literature. HPLC/APCI-MS analysis of acidic biomass hydrolysates suggests that the membrane of *M. sedula* strain TH2 mainly contains tetraether membrane lipids, however lacking information about associated headgroups (Hopmans et al. 2000; Meer et al. 2001).

In this study, we present the development of a defined medium for the heterotrophic cultivation of *M. sedula*. Beginning with evaluating different complex substrates, we deciphered the AA composition of CasA. Imitating the composition of CasA, we compared the specific substrate uptake rates of the individual AAs q_S, Amino Acid_ to reduce the number of components in the medium. Finally, MALDI-MS analysis of lipid extracts was performed to investigate the lipid composition of *M. sedula*. We believe that with the developed defined growth conditions, a more streamlined genetic manipulation using auxotrophies will be facilitated, and a big step towards more biotechnological applications, such as the production of unique ether lipids, was accomplished.

## Materials and methods

### Origin of complex medium sources

CasA (Lot# 5196721) was obtained from Thermo Fisher (USA). Casein Hydrolysate (Lot# 415231096), Yeast Extract (Lot# 173337187), and Tryptone (Lot# 147254688) were purchased from Carl Roth (Germany). N-Z-Amine (Lot# BCBZ4391) was obtained from Sigma-Aldrich (USA).

### Media preparation

Brock basal medium served as the mineral source as described earlier (Brock et al. 1972), with the exception of substituting equimolar amounts of FeCl_3_ with Fe(III)-citrate. Complex medium sources were used at a concentration of 1 g/L. To allow direct comparability and consistency across experiments, the total concentration of AAs in the defined media was 1 g/L. The initial pH of all cultivation media was set to pH = 2.2 using H_2_SO_4_ (4.8%).

### Cultivation conditions

#### Cultivation of *Metallosphaera sedula* in long neck shake flasks

*M. sedula* DSM 5348 was cultivated aerobically at 75 °C. To avoid evaporation and create a reflux-condensing environment, long neck shake flasks (100 mL) were used in a shaking oil bath (Memmert, Germany) with 50 mL culture medium to provide enough gas space for adequate oxygen supply. Separate precultures were used as inoculum for all growth experiments in this study. To prevent a carryover of medium components, the cell broth of the preculture was centrifuged (5,000 × *g*; 5 min), and the cell pellet was resuspended in 1 mL of fresh culture medium. Initial optical density after inoculation ranged between 0.005 and 0.01 in all experiments. All cultivations were carried out in triplicates.

#### Cultivation of *Metallosphaera sedula* on solid plates

Culture plates were prepared using Brock basal salts supplemented with 0.25 g/L CaCl_2,_ 0.5 g/L MgCl_2,_ and 0.6% (w/v) gelrite at pH = 3.0, as described earlier (Rastädter et al. 2022). The respective carbon source was added at a total concentration of 1 g/L. As an inoculum, cells in the exponential growth phase in liquid culture were diluted in Brock medium without carbon source to OD_600_ = 0.1 and further diluted 10^−5^ in the same medium. 50 µL of this suspension were pipetted onto the plates, dispersed, and then incubated at 75 °C for seven days.

### Analytical methods

#### *Determination of biomass concentration *via* optical density*

Optical density (OD_600_) was determined photometrically on an ONDA V-10 PLUS (Giorgio Bormac, Italy) at 600 nm against a blank of deionized water. Dry cell weight (DCW; [g/L]) was calculated by multiplication of the measured OD_600_ values with a factor of 0.171, which derived from an OD_600_: DCW correlation curve of *M. sedula* cells grown on Brock basal + 1 g/L Tryptone.

#### HPLC analysis for the detection of uracil

Standard solutions of Tryptone, Yeast Extract, CasA, N-Z-Amine, and Casein Hydrolysate were analyzed for their uracil content via HPLC measurement. Analysis was conducted on a Vanquish Core HPLC system using an Aminex HPX-87H column (300 × 7.8 mm; Bio-Rad, USA) employed with a pre-column (Micro-Guard Cation H + cartridge, 30 × 4.6 mm; BioRad). The column temperature was 60 °C, while the flow rate was 0.6 mL/min. Isocratic elution was achieved with 4 mM H_2_SO_4,_ and detection occurred via RI measurement on a RefractoMax 520 (IDEX Health & Science, USA). Analysis was conducted for three sample replicates per complex medium source.

#### HPLC analysis for the detection of amino acids

At each sampling point, 1 mL of culture broth was centrifuged at 10,000 × *g* and 4 °C for 10 min. The supernatant was analyzed for its AA composition via HPLC measurement on an Ultimate 3000 (Thermo Fisher Scientific, USA). Analyte separation was achieved with a reversed-phase column (Agilent ZORBAX Eclipse AAA; 150 × 3 mm, 3.5 µm; Agilent, USA) equipped with the pre-column (Agilent Eclipse AAA; 12.5 × 3 mm, 5 µm; Agilent) on an Ultimate 3000. The mobile phase comprised 40 mM NaH_2_PO_4_•H_2_O, pH = 7.8 (eluent A), and methanol:acetonitrile:mq = 45:45:10 (eluent B). The flow rate and the column temperature were 1.2 mL/min and 40 °C, respectively. After 2.5 min of equilibration at 100% A, a gradient was applied for 17.5 min to 48.5% buffer B. After a 3 min washing step at 100% B, the column was re-equilibrated to starting conditions for 2.5 min. For detection, an in-needle derivatization step was performed. For detection of primary amines, ortho-phtaldialdehyde containing 1% 3-mercaptopropionic acid, and for secondary amine detection, 9-fluormethylen-carbonylchloride was used as a derivatization agent. Norvaline (primary amines) and sarcosine (secondary amines) served as internal standards and were added with a final concentration of 1.25 mM to all samples and standards. Primary amines and norvaline were detected at 340 nm/450 nm (excitation/emission), and secondary amines and sarcosine were detected at 266 nm/305 nm (excitation/emission). Blank samples of medium without inoculum were analyzed to record changes in the AA composition caused by the incubation under the respective cultivation conditions.

#### ICP-OES analysis

ICP-OES analysis was conducted to determine the levels of contamination by common trace elements in tryptone and CAA. The following elements with their respective limit of detection (LOD) in parenthesis as µg/g were investigated: B (0.066), Ca (0.056), Co (0.119), Cu (0.161), Fe (0.093), K (1.125), Mg (0.002), Mn (0.016), Mo (0.181), V (0.044), and Zn (0.085). ICP-OES for simultaneous, multi-element analysis was carried out on an iCAP 6500 series spectrometer (Thermo Scientific) coupled to an ASX-520 autosampler (Teledyne Cetac, Omaha, NE, USA). The instrument had a standard sample introduction set consisting of a concentric nebulizer and a cyclonic spray chamber. Two sensitive and non-interfered emission lines per element were used, one for quantification and the second for quality control. Samples were stored at 4 ◦C until measurement. The sample solutions were diluted 1:10 with 1%-HNO_3_ (v/v) for matrix adjustment; thereby, Europium with a concentration level of 1.0 mg/L has been added as an internal standard to all samples. Quantification was done via external calibration with aqueous standard solutions using Europium as an internal standard. High-purity water was obtained by purifying deionized water with an Easypure 2 system (Thermo Scientific). Nitric acid was of p.a. grade purity (Merck, Germany). A certified stock solution of Europium (Specpure ICP standard; Alfa Aesar, Germany), Certipur multi-element VIII, and Molybdenum single element ICP-standard (Merck KGgA, Germany) were used for method development and quantification of sample signals. Before use, working solutions were prepared by diluting stock solutions with 1.0% nitric acid (v/v).

#### Lipid extraction, sample preparation and reflectron MALDI time-of-flight mass spectrometry analysis

Lipid extraction was performed as previously described (Quehenberger et al. 2020). In short, 50 mL of cell suspension was harvested in the exponential phase and centrifuged at 4000 × *g* for 5 min. Subsequently, the cell pellet was resuspended in a cold solution of 155 mM ammonium acetate and lysis performed via sonication (Branson 450 digital sonifier, Branson Ultrasonics, USA; 50% amplitude, 30 s on 30 s off, 5 min total) on ice. The lysate was then diluted to reach an OD_600_ value of 0.5, then mixed with a mixture of chloroform:methanol (2:1, v/v; Folch solution). After shaking for 4 h at 300 rpm at 4 °C and a centrifugation step (1000 × *g*, 2 min, 4 °C), the lower organic phase was collected and the solvent was removed using a rotary evaporator (R-210, Büchi, Switzerland). Archaeal lipid extracts were stored at – 20 °C until measurement. All archaeal lipid fractions were dissolved with Folch solution at 1 g/L. For analysis. 2-,4-,6-Trihyroxy-acetophenone (Sigma-Aldrich, USA) was selected as the MALDI-matrix for all experiments by dissolving roughly 20 mg of matrix in 1 mL methanol. For final sample preparation, matrix and analyte solution were mixed 1:1 (vol/vol) immediately before applying 0.5 µL of the resulting mixture onto a standard stainless-steel target surface. All measurements were performed using a Bruker Daltonics ultrafleXtreme reflectron MALDI time-of-flight mass spectrometer (Bruker Daltonics, Germany) fitted with a frequency-tripled 2 kHz Nd-YAG laser (λ = 355 nm) typically operated at a repetition rate of 200 Hz. The accelerating voltage was set to ± 20 kV for positive- and negative-ion detection, respectively. All MALDI mass spectra are acquired in the positive- (detected ions: [M + Na]^+^ and [M + 2Na − H]^+^) and negative-ion (detected ions: [M − H]^−^) reflectron TOF–MS mode, respectively (average of 10.000 spectra). For positive- and negative-ion mass calibration, a commercial peptide mix (supplied by Laser Biolabs, Valbonne, France) based on peptides of known mass values prepared on α-cyano-4-hydroxy cinnamic acid (CHCA) matrix was used.

### Calculation of specific rates

#### Specific substrate uptake rates

The specific substrate uptake rate for the individual amino acids, q_s, Amino Acid_ [g/g/h], between two sampling points was calculated via the following equation:

$${q}_{S, Amino Acid}=\frac{{c}_{Amino Acid, t2}-{c}_{Amino Acid, t1}}{\overline{x }*({t}_{2}-{t}_{1})}$$

c_Amino Acid, t2_ is the concentration of the individual amino acid at the sampling time t_2_, c_Amino Acid, t1_ is the concentration of the individual amino acid at the sampling point t_1_. x̄ is the mean DCW value of the two sampling points. The provided values for q_s_ correspond to the mean of the individual specific substrate uptake rates for all sampling points during the exponential growth phase until substrate depletion.

#### Specific growth rate

The specific growth rate, µ [h^−1^], between two sampling points was calculated via the following equation

$$\mu =\frac{ln\frac{{x}_{2}}{{x}_{1}}}{{t}_{2}-{t}_{1}}$$

x_2_ is the DCW value at sampling point t_2_, x_1_ is the DCW value at the sampling point t_1_. The provided values for µ correspond to the mean of the individual specific growth rates for all sampling points during the exponential growth phase.

## Results and discussion

### Uracil content in complex substrates

Various complex substrates (Tryptone, Beef Extracts, Yeast Extract, N-Z-Amine, CasA, and Casein Hydrolysate) have been employed in the literature to allow heterotrophic cultivations of *M. sedula* (Huber et al. 1989; Auernik and Kelly 2010b; Maezato et al. 2012). Apart from oligopeptides and AAs, complex substrates incorporate salts, nucleotides, and components, which may act as inhibiting agents during microbial cultivation. The presence of uracil in complex media has been associated with unwanted background growth and selection of false positives using uracil auxotrophy as a selection marker of the genetically engineered *M. sedula* strain PBL4001 (McCarthy et al. 2018). Uracil auxotrophy is a commonly used selectable marker employed for *Sulfolobales* species. Usually, these strains contain mutations in genes coding for orotidine pyrophosphorylase (*pyrE*) and/or orotidine decarboxylase (*pyrF*). Integration of the gene of interest recovers the ability of uracil biosynthesis; however, it requires maintaining a stringent selection condition (Wagner et al. 2012). The consensus remains that uracil-based selection cannot be employed efficiently in *Sulfolobales*, partly due to uracil traces in the substrate used on solid media, which cause interferences by background growth (Zhang et al. 2013). Using HPLC analysis, we analyzed commonly used complex substrates for their uracil content (Table 1). Traces of uracil were detected in all analyzed substrates. The uracil content determined for Tryptone and Yeast Extract was in a concentration range, posing a risk of false positive selection for uracil auxotrophic *Sulfolobales* strains. While strong growth was observed for uracil concentrations of 10–25 µg/mL, auxotrophic *Sulfolobus acidocaldarius* strains KH1U, KH2U, and MW001 grow already at concentrations of 1 µg/mL (Choi and Cha 2011; Wagner et al. 2012; Ye et al. 2022).

**Table 1:** Uracil content of complex C-sources in µg/g used for heterotrophic cultivation of *M. sedula*

Substrate	Casamino Acids	Yeast extract	Tryptone	N-Z-Amine	Casein hydrolysate
Uracil content [µg/g]	0.042 ± 0.01	1.05 ± 0.01	0.091 ± 0.02	0.026 ± 0.001	0.0016 ± 0.0002

### Growth kinetics of *Metallosphaera sedula* on commonly employed complex substrates

**Fig. 1:**
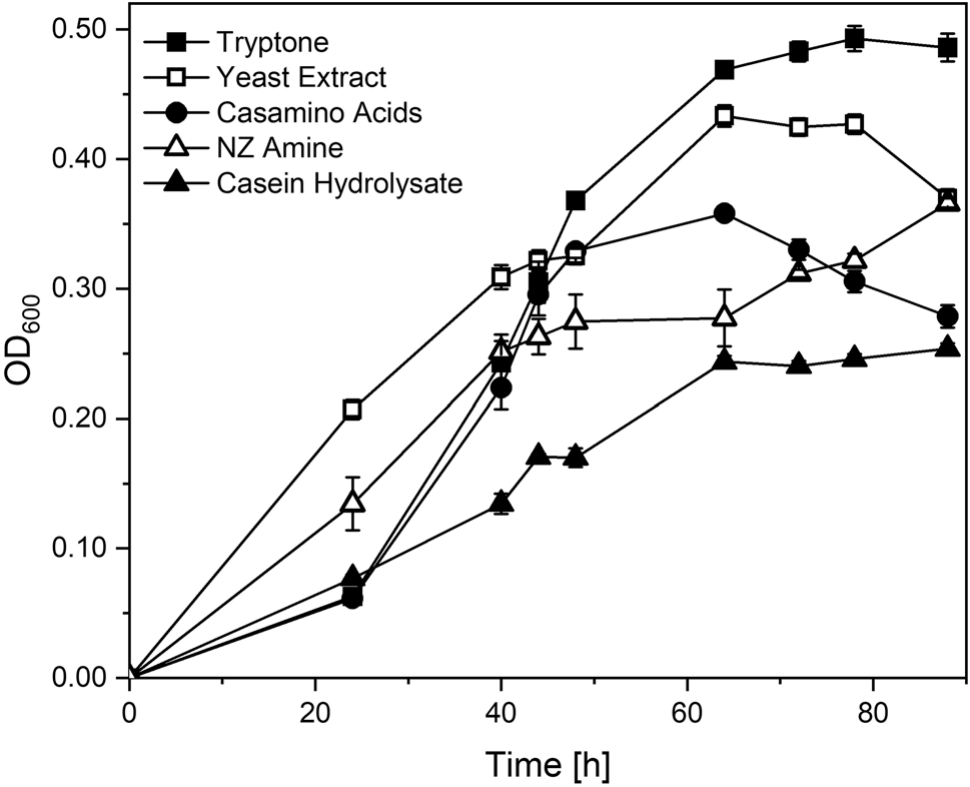
Growth kinetics of *Metallosphaera sedula* on Tryptone (Black square), Yeast Extract (White medium square), Casamino Acids (Black circle), N-Z-Amine (White Up-Pointing triangle) and Casein Hydrolysate (Black up-Pointin triangle) (at a concentration of 1 g/L in Brock basal medium at pH = 2.2 and 75 °C. Values for OD_600_ are represented as mean ± standard deviation and derive from biological triplicates

So far, efforts to develop defined conditions for cultivating *M. sedula* have been unsuccessful. As it is known that *M. sedula* is incapable of metabolizing sugars (Huber et al. 1989), our strategy was to find a suitable AA-based complex substrate that facilitates strong cell growth and contains a high degree of free AA, enabling accessibility to standard analytical practices. Figure 1 displays the growth kinetics of *M. sedula* on commonly used complex substrates.

The highest biomass concentration after 88 h was achieved using Tryptone, followed by Yeast Extract. Both complex substrates have been the golden standard for heterotrophic cultivations, most likely due to the cell densities achieved using these complex medium sources (Auernik et al. 2008; Auernik and Kelly 2010b). Tryptone is derived from enzymatically treated casein and primarily contains oligopeptides (Puhm et al. 2022). Likewise, Yeast Extract originates from the mild treatment of biomass and consists of larger polypeptides (Leong et al. 2016; La Rosa et al. 2016). The high share of polypeptides, however, restricts our criterion for rather simple analytics. Reports indicate that the mass fraction of free amino acids in Tryptone and Yeast Extract falls between 25 and 35%. (Armstead and Ling 1991; Xu et al. 2020; Tomé 2021).

On CasA, exponential growth could be observed, reaching a maximum after 62 h (OD_600_ = 0.36). For N-Z-Amine, biomass concentration followed a more linear trend, achieving similar optical densities only after 88 h. Cultivations on Casein Hydrolysates reached stationary phase after 62 h at OD_600_ = 0.24. Like Tryptone, CasA, N-Z-Amine, and Casein Hydrolysate are products of treated casein. While N-Z-Amine is a product of enzymatically treated casein with a subsequent refining step, CasA and Casein Hydrolysate are subject to acidic hydrolysis of casein. This manufacturing process, using harsh conditions of acidic treatment, increases the content of free AAs in the final extract since peptide bonds are cleaved quantitively compared to the treatment with proteases. As CasA supports the strongest growth among the three, we based our further development on this AA source.

### HPLC analysis of amino acids composition in Casamino Acids

HPLC analysis was performed to quantify the content of free AAs in CasA (Table 2). Apart from glutamine, asparagine, tyrosine, and tryptophan, 16 AAs were detected. Glutamine and asparagine are transaminated and converted to glutamate and aspartate, respectively, during the manufacturing of CasA. Likewise, the production process's acidic conditions cause tyrosine and tryptophan degradation (Fountoulakis and Lahm 1998).

**Table 2:** Free amino acid content of Casamino Acids (in milligrams per gram Casamino Acids) and their proportion of total free Amino Acid content (in %).

Amino acid	Free amino acid content [mg/g]	Proportion of total free amino acids [%]
Glutamate	154.3 ± 2.3	21.8 ± 0.3
Proline	94.4 ± 3.2	13.4 ± 0.4
Lysine	65.7 ± 4.6	9.3 ± 0.7
Aspartate	51.5 ± 1.0	7.3 ± 0.1
Leucine	43.9 ± 0.3	6.2 ± 0.0
Valine	40.1 ± 2.2	5.7 ± 0.3
Serine	38.8 ± 1.1	5.5 ± 0.2
Cysteine	29.0 ± 4.0	4.1 ± 0.6
Threonine	28.6 ± 0.4	4.1 ± 0.1
Arginine	26.7 ± 1.4	3.8 ± 0.2
Isoleucine	26.5 ± 0.8	3.8 ± 0.1
Phenylalanine	26.5 ± 5.1	3.8 ± 0.7
Alanine	24.3 ± 0.3	3.4 ± 0.0
Methionine	23.3 ± 3.0	3.3 ± 0.4
Histidine	18.0 ± 0.5	2.6 ± 0.1
Glycine	14.6 ± 0.5	2.1 ± 0.1

Four proteinogenic amino acids (tryptophan, tyrosine, glutamine, and asparagine) are deaminated or degraded during the acidic hydrolysis

It is challenging to contextualize the results within the literature, as previous analyses of CasA either lacked information about single AAs due to analytical limitations (D’Huys et al. 2011) or were based on theoretical calculations (Neumann-Schaal et al. 2015). Additionally, the variability arising from biologically sourced materials and the fluctuating batch-to-batch composition are inherent. A detailed study conducted by Wang et al. showed variations of, e.g., glutamate content between 5 different acidic casein hydrolysates of 60% (Wang et al. 2016). However, an increased cysteine content is apparent in our analysis. While the literature reports a cysteine content in casein hydrolysates ranging from 0.1% up to 1% (w/w) (Rasmussen 2008; Neumann-Schaal et al. 2015), we could find a share of 4.1% of total free AAs. These elevated levels might stem from competitive reactions with the derivatization reagents necessary for fluorescence detection or the general poor fluorescence signal for this analyte (Rawat and Maupin-Furlow 2020). Additionally, in line with existing literature, glutamate and proline constitute the predominant AAs (Rasmussen 2008; Wang et al. 2016). Total free AA content accounted for ~ 71% of the total mass of CasA. The remaining share likely comprises oligo- and polypeptides not cleaved during hydrolysis. ICP-OES analysis of selected trace elements in CasA revealed a total content of less than 1 mg/g (Supplementary Table S1).

### Cultivation of *Metallosphaera sedula* on amino acid composition of Casamino Acids

**Fig. 2:**
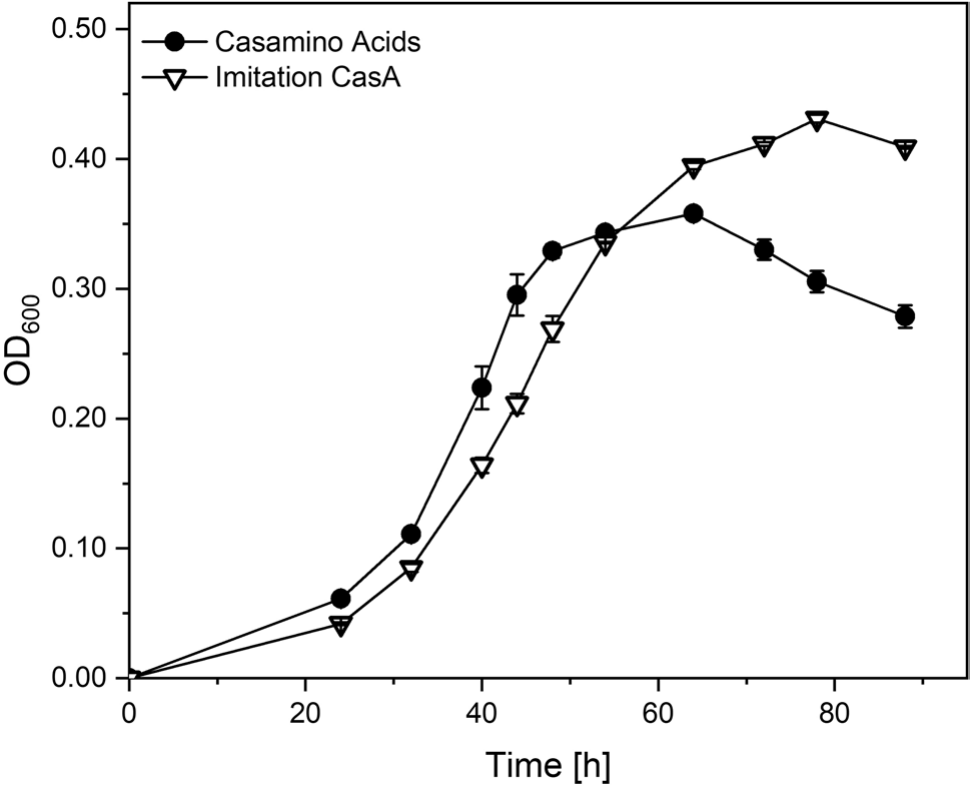
Growth kinetics of *Metallosphaera sedula* on Casamino Acids (Black circle) and imitation of Casamino Acids (Imitation CasA) (White Down-pointing Triangle) at a concentration of 1 g/L in Brock basal medium at pH = 2.2 and 75 °C. Values for OD_600_ are represented as mean ± standard deviation and derive from biological triplicates

In the next step and with the knowledge of the AA composition of CasA, we performed shake flask experiments, mimicking the AA concentrations of CasA. Figure 2 compares the growth kinetics of *M. sedula* cultivated on CasA and the imitation. Similar growth curves can be observed for both media until 44 h of cultivation time, when cells grown on CasA reached the plateau of stationary phase at OD_600_ = 0.33, while cells cultivated on the imitation reached the stationary phase after 72 h at OD_600_ = 0.43. This substantial increase in biomass can be rooted in several factors. The unknown proportion of polypeptides in CasA restricts a comparison of cultivations based on molar carbon content in the two media. Accordingly, molar carbon content might be higher in the imitation than in CasA, allowing the conversion of more substrate into biomass. However, as the biomass concentration is boosted by ~ 25% in the imitation, this observation likely relies on more efficient utilization of free AAs than polypeptides in CasA. For both cultivation conditions, all AAs were taken up quantitatively by the end of the experiment at 96 h. The specific growth rates of *M. sedula* cultivated on CasA and the imitation, calculated as the mean of sampling points during the exponential growth phase between 32 and 44 h, were µ_CasA_ = 0.077 h^−1^ and µ_imitation_ = 0.076 h^−1^, respectively.

**Fig. 3:**
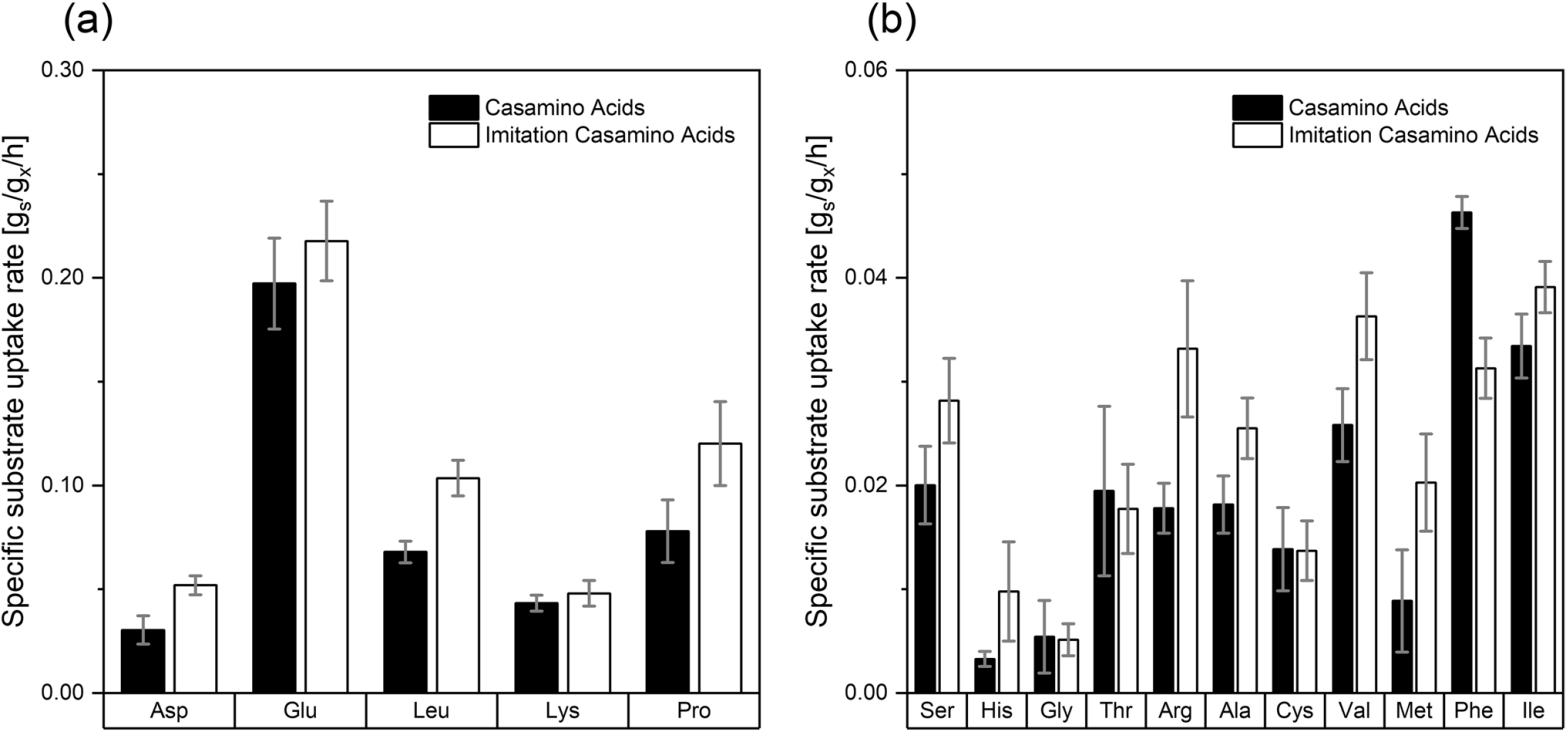
Comparison of substrate uptake rates for the single amino acids (q_s, amino acid_) of of *Metallosphaera sedula* cultivated on Brock basal supplemented with 1 g/L Casamino Acids (black) and 1 g/L imitation of Casamino Acids (white) at pH = 2.2 and 75 °C. (**a**) q_s_ for: Asp aspartate, *Glu* glutamate, *Leu* leucine, *Lys* lysine, *Pro* proline, (**b**) q_s_ for: Ser serine, His histidine, *Gly* glycine, *Thr* threonine, *Arg* arginine, *Ala* alanine, *Cys* cysteine, *Val* valine, *Met* methionine, *Phe* phenylalanine, *Ile* isoleucine, values are represented as mean ± standard deviation and were calculated for exponential growth phase between 24 and 44 h before substrate depletion from three replicates for every cultivation condition

We calculated substrate uptake rates q_S, Amino Acid_ for these cultivations (Fig. 3). Similar rates can be observed for the two media. Glutamate and proline, the AAs present at the highest concentration, were taken up at the highest rate, followed by leucine, aspartate, lysine, isoleucine, valine, arginine and phenylalanine. Phenylalanine displays the only AA for which a higher q_S, Amino Acid_ was determined for cultivation on CasA. It can be speculated, that increased substrate uptake rates for individual amino acids rely on the additional presence and utilization of polypeptides in CasA.

### Amino acids essential for heterotrophic cultivation of *M. sedula*

**Fig. 4:**
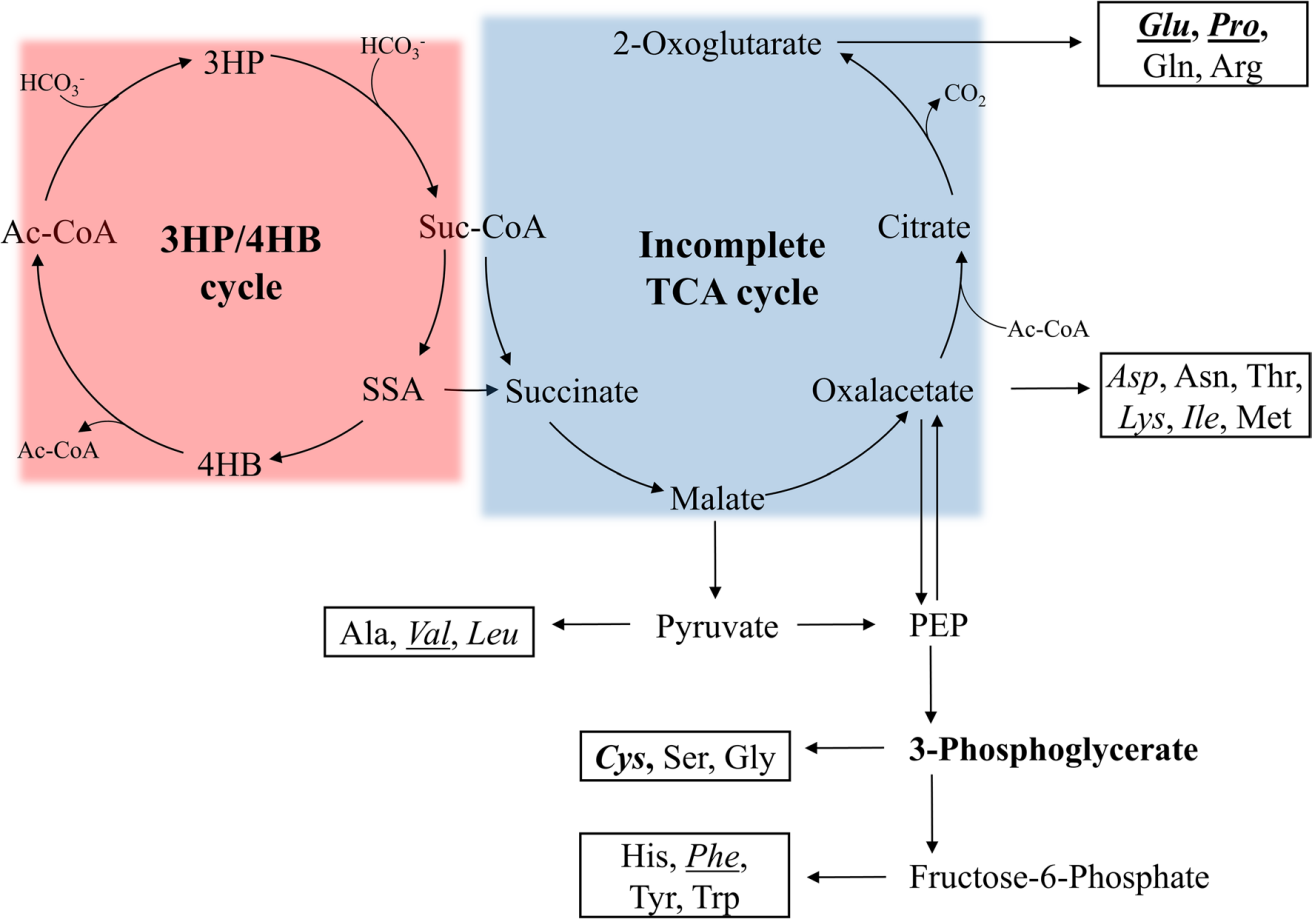
Proposed central metabolism in *Metallosphaera sedula,* including 3-Hydroxypropionate/ 4-Hydroxybutyrate cycle (3HP/4HB cycle) (red, left), incomplete tricarboxylic acid cycle (blue, center) and precursors for amino acid biosynthesis. Essential amino acids are highlighted in bold, amino acid components of the 5AA medium are in italics and amino acid components of the PS medium are underlined. Abbreviations in alphabetical order: *Ac-CoA* acetyl-coenzyme A, *Ala* alanine, *Arg* arginine, *Asn* asparagine, *Asp* aspartate, *Cys* cysteine, *Gln* glutamine, *Glu* glutamate, Gly glycine, *His* histidine, *3HP* 3-hydroxypropionate, *4HB* 4-hydroxybutyrate, *Leu* leucine, *Lys* lysine, *Ile* isoleucine, *Met* methionine, *PEP* phosphoenolpyruvate, *Phe* phenylalanine, *Pro* proline, *Ser* serine, *SSA* succinyl semialdehyde, *Suc-CoA* succinyl-coenzyme A, *Thr* threonine, *Trp* tryptophan, *Tyr* tyrosine, *Val* valine

In the following experiments, the ratio of the individual AAs was set based on their occurrence found in CasA and normalized to a total AA concentration of 1 g/L. Initial screening experiments enabled us to categorize the AAs into two groups: A.) Essential constituents critical for cell growth (glutamate, proline, cysteine), and B.) Potentially growth-boosting constituents, facilitating cell growth. Cultivations with only the three essential AAs resulted in an OD_600_ of only 0.12 after 92 h (Supplementary Information Fig. S1), limiting strong growth on solid plates. The composition of this medium can be found in the Supplementary Information Table S1. Although the genome of *M. sedula* has been sequenced and several transcriptome analysis studies have been conducted, the current understanding of its central carbon metabolism in heterotrophy fails to explain this observation adequately. The absence of 2-oxoglutarate dehydrogenase prevents the conversion of 2-oxoglutarate to succinyl-CoA, thus rendering the TCA cycle incomplete in *M. sedula* (Fig. 4). Additionally, the relevance of the glyoxylate cycle remains unclear, as neither isocitrate lyase activity nor its putative gene was found in transcriptome/ genome analysis of heterotrophically grown cells (Estelmann et al. 2011). Proline and glutamate are both synthesized from and can be converted to 2-oxoglutarate. Accumulated 2-oxoglutarate, due to the missing dehydrogenase, is depleted via arginine, glutamine, proline, and glutamate biosynthesis. Our observation, however, suggests that 2-oxoglutarate may also act as a central metabolite and that carbon flux can be generated from glutamate and proline. This could be explained by a significantly active ferredoxin-dependent oxidoreductase, converting 2-oxoglutarate to succinyl-CoA, a hypothesis already raised by Estelmann et al. (2011). Archaeal ferredoxin-dependent oxidoreductases have been shown to possess a broad substrate specificity (Park et al. 2006). The regenerated succinyl-CoA, obtaining a pivotal role during autotrophic carbon fixation of *M. sedula* (Fig. 4), can again act as a building block for all precursors for AA synthesis. The necessity of cysteine can be explained by the burden of limited reducing power caused by the incomplete TCA and glyoxylate cycle. In chemolithoautotrophic conditions, this shortage is compensated through supplies of reducing equivalents via iron and metal sulfide oxidation. Insufficient reducing power might hinder the reduction of sulfate (only sulfur-containing compound in Brock basal medium) to sulfide, essential for cysteine biosynthesis. This circumstance might necessitate external supply during heterotrophic cultivation. Replacing cysteine with methionine did not lead to any biomass growth.

### Screening for growth-boosting amino acids for heterotrophic cultivation of *M. sedula*

**Fig. 5:**
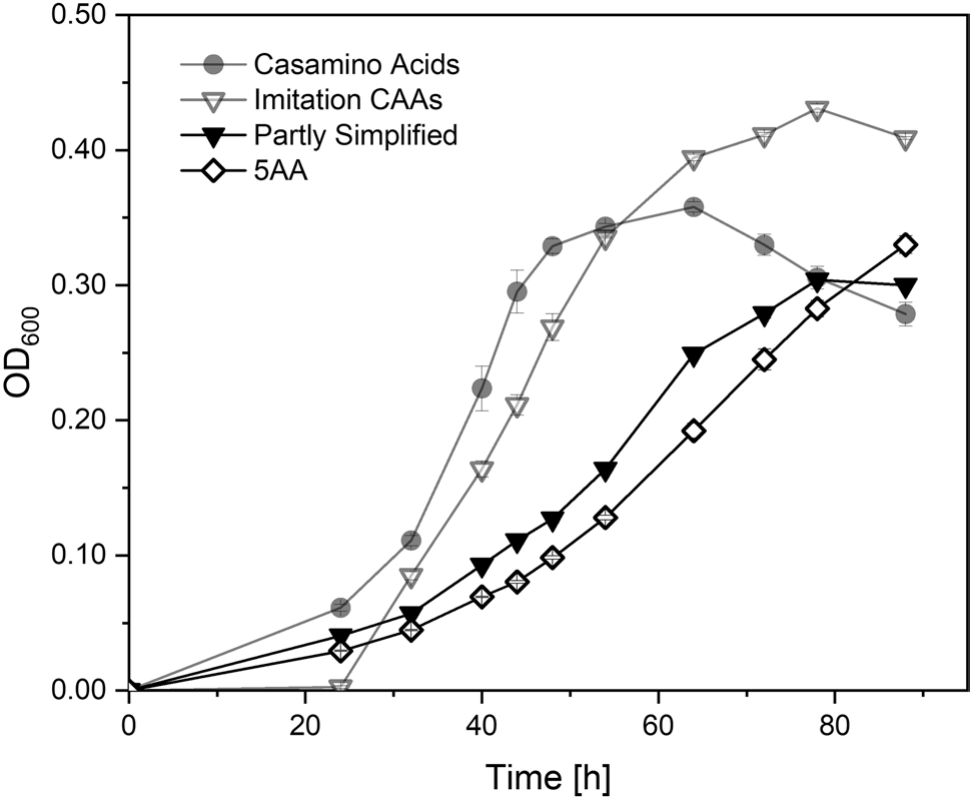
Growth kinetics of *Metallosphaera sedula* on Partly Simplified (Black down-Pointin triangle ) and 5AA medium (White Diamond) at a concentration of 1 g/L in Brock basal medium at pH = 2.2 and 75 °C. Growth kinetics of Casamino Acids (Black circle) and Imitation of Casamino Acids (Imitation CasA) (Black up-Pointin triangle), as seen in Fig. 2, are plotted transparently for better comparability. Values for OD_600_ are represented as mean ± standard deviation and derive from biological triplicates

As our goal was to develop a medium that allows cell growth similar to the complex state-of-the-art media and efficiently promotes growth on solid plates, we investigated the addition of other AAs. We only included AAs with a q_s, Amino Acid_ > 0.03 g/g/h for further screenings to minimize the number of experiments. Figure 5 shows the growth kinetics of *M. sedula* cultivated on the "partly simplified" (PS) medium, incorporating 9 AAs with.

q_s, Amino Acid_ > 0.03 g/g/h, and cysteine as an essential AA. After 78 h, an OD_600_ of 0.31 could be achieved using the PS medium, exposing an extended lag phase compared to the imitation of CasA and complex media. Subsequently, we tried to identify strong growth-promoting AAs, performing several screening experiments adding single and combinations of AAs with q_s_ > 0.03 g/g/h. The total AA concentration during these screenings was kept at 1 g/L, retaining the ratios of the individual AA as they were found in CasA. The results of the screening experiments can be found in the Supplementary information (Supplementary Fig. S1 and Supplementary Fig. S2). We found phenylalanine and valine to boost cell growth most substantially. Compared to growth glutamate, proline and cysteine alone, where OD_600_ of 0.12 was achieved after 88 h, cell densities of OD_600_ = 0.27 (phenylalanine) and OD_600_ = 0.30 (valine) were observed. The growth kinetics of *M. sedula* cultivated on the combination of phenylalanine, valine, and the essential AAs can be seen in Fig. 5. We named this medium 5AA, and the exact composition can be found in Table 3. Similar to PS, an extended lag phase was observed, however reaching a similar optical density of 0.33 with similar kinetics (µ_PS_ = 0.049 h^−1^; µ_5AA_ = 0.048 h^−1^), demonstrating that the reduction to only 5 AAs components does not affect cell growth. Further cultivations of *M. sedula* on solid plates confirmed the applicability of this novel cultivation medium (Supplementary Fig. S4).

**Table 3:** Amino acid composition of the 5AA medium

Amino acid	Concentration [mg/L]
Glutamate	400
Proline	290
Cysteine	150
Valine	110
Phenylalanine	50

### Reflectron MALDI TOF–MS lipid analysis of *M. sedula* cultivated on defined medium

**Fig. 6:**
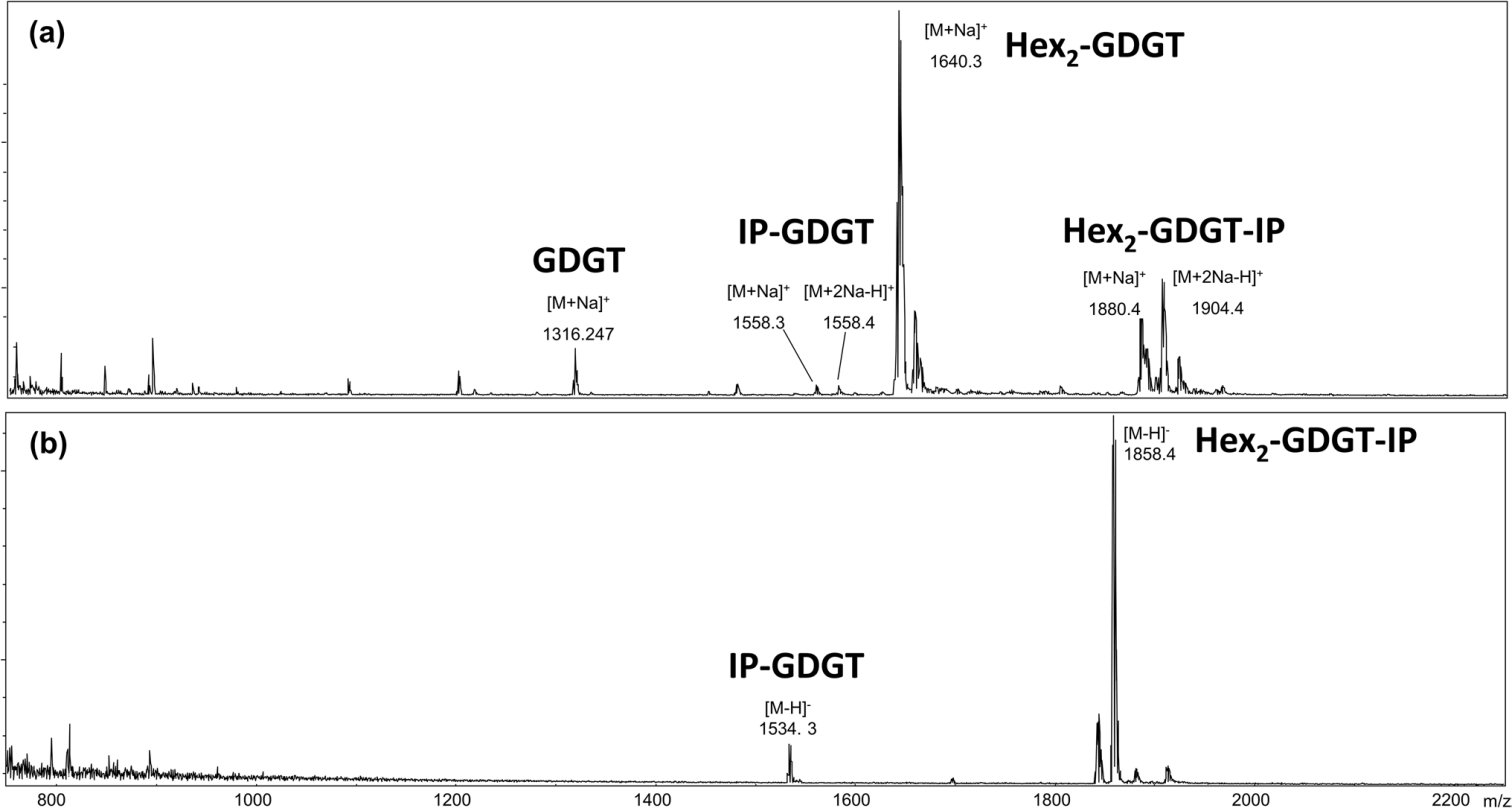
Reflectron MALDI TOF–MS spectrum of *Metallosphaera sedula* lipid extract cultivated on 5AA medium in (**a**) positive-ion mode and (**b**) negative-ion mode

We further performed MALDI-MS analysis of lipid extracts from *M. sedula* biomass cultivated on the defined 5AA medium. Stable membrane lipids, characterized by isoprenoid chains connected to a glycerol via ether bonds in the *sn*-2,3 position, are characteristic of archaea. They can be classified into two classes: C_20_ isoprenoid chain containing diether lipids (DGD) and C_40_ containing isoprenoid chain containing tetraether lipids (GDGT), which can further be modified with various headgroups, most commonly hexose(s) (Hex) or inositol phosphate (IP). Due to their stability attributes, archaeal membrane lipids have been investigated for their utilization as lipid excipients for oral drug delivery (e.g. (Quehenberger et al. 2017; Uhl et al. 2021; Sedlmayr et al. 2023)). Recently, we have shown the effect of tetraether lipids on endosomal escape, revealing their promising application in next-generation lipid nanoparticle formulations for mRNA delivery (Sedlmayr et al. 2024). So far, an HPLC/APCI-MS analysis of *M. sedula* biomass extracts revealed a high content of tetraether lipids, but a more detailed analysis of the lipid composition was missing. Since extraction of the lipids was conducted under acidic conditions, resulting in the cleavage of the polar headgroups (Hopmans et al. 2000). To analyze the lipid composition of *M. sedula* in more detail*,* we performed a modified Folch extraction as described earlier (Quehenberger et al. 2020). Figure 6 displays the MALD-MS spectrum of the extract obtained from the biomass grown on the defined 5AA medium. The most abundant lipid classes we identified were Hex_2_-GDGT, Hex_2_-GDGT-IP, GDGT, and IP-GDGT. Other lipid classes were excluded from the analysis due to their sporadic appearance around noise levels. No diether lipid species could be detected, confirming Hopmans et al. assumption that *M. sedula* mainly contains tetraether lipid species.

Table 4 shows the relative abundance of the four most prominent lipids detected in *M. sedula*. The highest abundance was found for Hex_2_-GDGT, while the relative abundance of Hex_2_-GDGT-IP was 27%. Similar analyses of a related organism have only been conducted with different *Sulfolobus* spp, where diether lipids constitute a substantial proportion of the total lipid composition. Depending on the growth conditions, *Sulfolobus* spp. contain 15–50% diether lipids (Jensen et al. 2015a, b; Quehenberger et al. 2020). Also, elevated levels of Hex_2_-GDGT-IP can be recognized for *M. sedula*, which only comprises roughly 7% of tetraether lipids in *S. acidocaldarius* (Quehenberger et al. 2020).

**Table 4:** Relative abundance of most prominent lipids obtained in positive-ion MS mode for *Metallosphaera sedula* cultivated on the 5AA medium.

Lipid class	Relative abundance
GDGT	4 ± 2%
IP-GDGT	5 ± 2%
Hex2-GDGT	64 ± 5%
Hex2-GDGT-IP	27 ± 5%

Values are represented as mean ± standard deviation determined by measuring five biological replicates

## Conclusion

Defined media facilitate laboratory work and fulfill the regulatory requirements of bioprocesses. In the case of *M. sedula*, the absence of a defined medium has complicated genetic engineering efforts, particularly concerning uracil auxotrophies. In this study, we presented a strategy that allowed us to find defined conditions for cultivating *M. sedula*. By carefully performing screening experiments, we found three essential amino acids necessary for biomass growth (glutamate, proline, and cysteine). This discovery suggests significant carbon flux originating from 2-oxoglutarate, which might be depleted via a highly active ferredoxin-dependent oxidoreductase, enabling the conversion of 2-oxoglutarate to succinyl-CoA. To boost cell growth with a minimal number of amino acids, we ended up with the defined "5AA" medium containing glutamate, proline, cysteine, phenylalanine, and valine. Although coming with an increased lag phase, the 5AA medium enables the cultivation to optical densities similar to CasA and allows for cultivations on solid media. Following our strategy, we believe that defined conditions for cultivating other *Sulfolobales* species can also be obtained. Moreover, we successfully used our novel defined cultivation medium to produce and analyze *M. sedula's* unique lipids. Reflectron MALDI TOF–MS analysis of heterotrophically cultivated *M. sedula* revealed a high content of tetraether lipids, while no diether derivates were detected. Tetraether lipids are currently under investigation for their utilization as lipid excipients for drug delivery, highlighting *M. sedula* as a promising production host. We envision the utilization of the 5AA medium in mixotrophic cultivations. Previous studies have demonstrated that combining CO_2_ and an organic source boosts the biomass growth of *M. sedula* (Auernik and Kelly 2010b).

## Supplementary Information

Below is the link to the electronic supplementary material.

Supplementary file1 (PDF 361 KB)

## Data Availability

All experimental data are available upon request.

## Abbreviations

AA: Amino acids
CasA: Casamino Acids
