# Supplementary material for: Development of a defined medium for the heterotrophic cultivation of Metallosphaera sedula
Source: Extremophiles. 2024 Jul 26;28(3):36. doi: 10.1007/s00792-024-01348-0 (PMC11282131; doi:10.1007/s00792-024-01348-0)
Supplement: Supplementary file 1 — Supplementary file1 (PDF 361 KB) [file 792_2024_1348_MOESM1_ESM.pdf]

Development of a defined medium for the heterotrophic cultivation of *Metallosphaera sedula*

Viktor Laurin Sedlmayr[1], Maximilian Luger[1], Ernst Pittenauer[2], Martina Marchetti-Deschmann[2], Laura Kronlachner[2], Andreas Limbeck[2], Philipp Raunjak[1], Julian Quehenberger[1] and Oliver Spadiut[1*]

[1]TU Wien, Institute of Chemical, Environmental and Bioscience Engineering, 1060 Vienna, Austria
[2]TU Wien, Institute of Chemical Technologies and Analytics, 1060 Vienna, Austria

* Corresponding author at: Institute of Chemical, Environmental and Bioscience Engineering, TU Wien, Vienna
E-mail address: oliver.spadiut@tuwien.ac.at (O. Spadiut)

Supplementary Information

Supplementary Table S1 ICP-MS analysis (in milligram per gram Tryptone/ Casamino Acids) of Mg, Ca, V, Mn, Fe, Sr, Zn, Na, K, B, Co and Cu in Tryptone and Casamino Acids. Co (LOD=0.119 µg/g) and Cu (LOD=0.161 µg/g) were below limit of detection (LOD).

**Element**	**Concentration in Casamino Acids [µg/g]**	**Concentration in Tryptone [µg/g]**
Mg	6.4	278.7
Ca	78.3	313.4
V	3.5	5.3
Mn	0.4	2.1
Fe	1.2	18.2
Sr	0.4	1.9
Zn	12.3	63.5
Na	188.5	102.6
K	58.3	133.5
B	0.202	0.232
Co	<LOD	<LOD
Cu	<LOD	< LOD

Supplementary Table S2

**Amino Acid**	**Concentration [mg/L]**
Glutamate	555
Proline	340
Cysteine	105

Supplementary Fig. S1

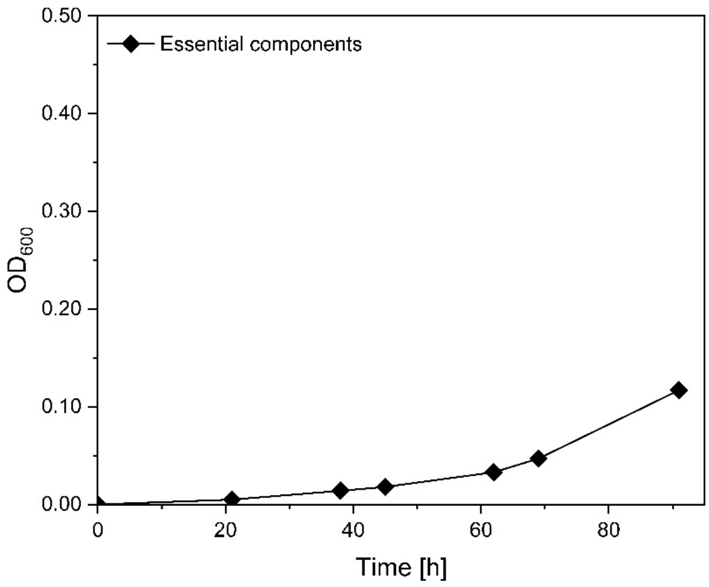

Supplementary Fig. S1 Growth kinetics of *M. sedula* DSM 5348 on Brock basal medium containing 555 mg/L glutamate, 340 mg/L proline and 105 mg/L cysteine at pH=2.2 and 75 °C.

Supplementary Fig. S2

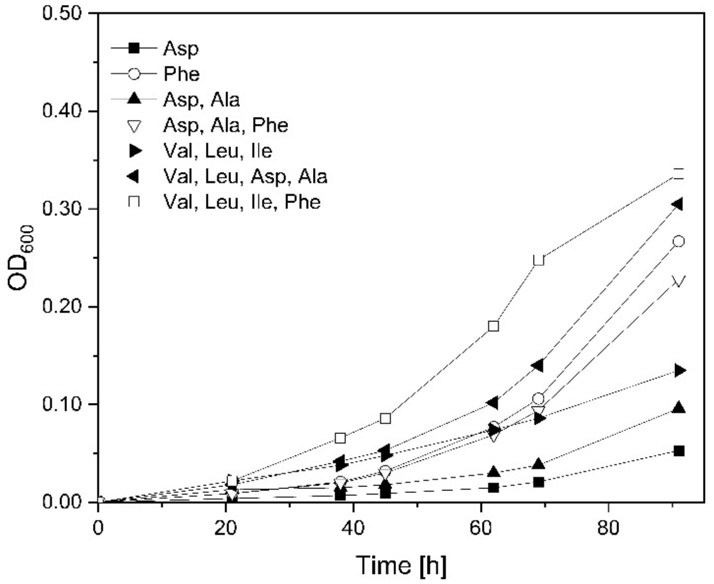

Supplementary Fig. S2 Screening experiments performed on the essential components supplied with either aspartate (Asp), phenylalanine (Phe), alanine (Ala), isoleucine (Ile); growth promoting effect of phenylalanine can be seen.

Supplementary Fig. S3

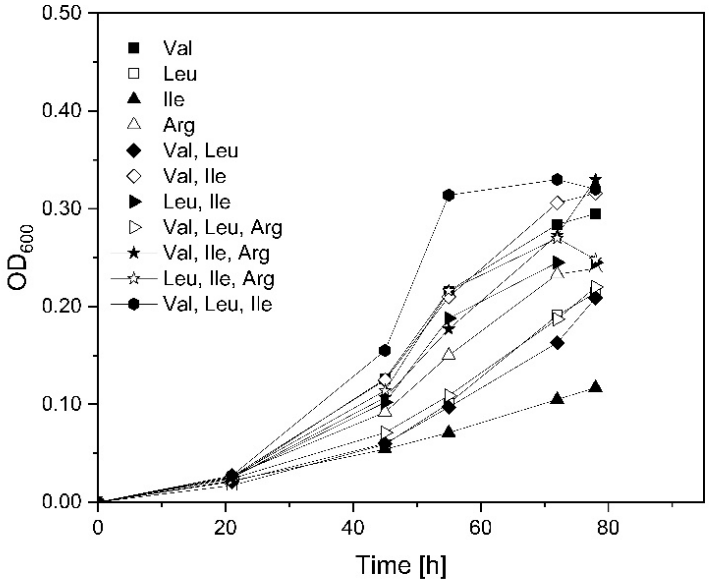

Supplementary Fig. S3 Screening experiments performed on the essential components supplied with either valine (Val), leucine (Leu), isoleucine (Ile), arginine (Arg), or a combination. Growth promoting effect of valine can be seen.

Supplementary Fig. S4

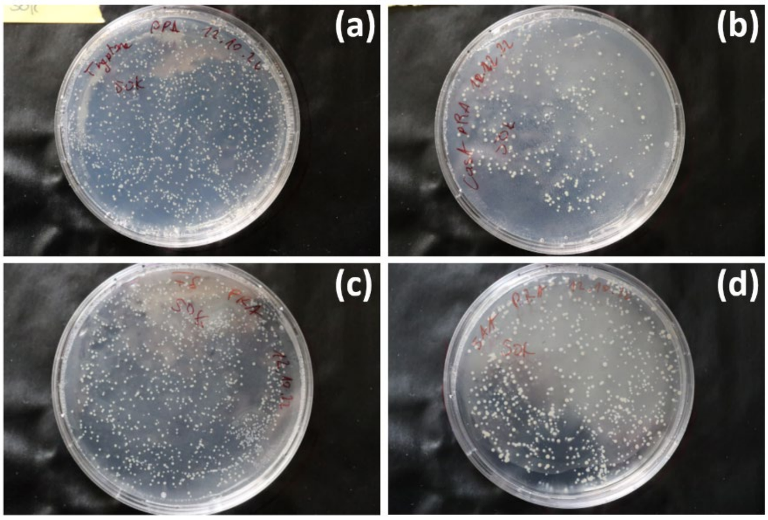

Supplementary Fig. S4 Cultivation of *Metallosphaera sedula* on solid plates using Tryptone (A), Casamino Acids (B), imitation of Casamino Acids (C), and the 5AA medium (D) as a medium component in Brock basal and gelrite-containing medium at pH= 3.0 after seven days of incubation at 75 °C.
